# Prevalence and associated factors for needlestick and sharp injuries (NSIs) among dental assistants in Jeddah, Saudi Arabia

**DOI:** 10.1186/s12199-019-0815-7

**Published:** 2019-10-10

**Authors:** Lama AlDakhil, Nagarajkumar Yenugadhati, Ohoud Al-Seraihi, Mustafa Al-Zoughool

**Affiliations:** 1 King Abdullah International Medical Research Center (KAIMRC), Riyadh, Kingdom of Saudi Arabia; 2 Department of Community and Environmental Health, College of Public Health and Health Informatics, King Saud bin Abdulaziz University for Health Sciences, Mail Code 2350, P.O. Box 22490, Riyadh, 11426 Kingdom of Saudi Arabia; 3 College of Dentistry, King Abdulaziz University, Jeddah, Kingdom of Saudi Arabia

**Keywords:** Healthcare workers, Dental assistants, Needlestick and sharp injuries, Infection control, Saudi Arabia

## Abstract

**Background:**

Dental personnel are subject to exposure to a number of occupational factors including needlestick and sharp injuries (NSIs). Our study aims to address knowledge gaps on prevalence and associated factors for needlestick and sharp injuries (NSIs) for the first time in Saudi Arabia.

**Methods:**

This cross-sectional study was conducted on a sample of 450 dental assistants recruited from 40 randomly selected private clinics in Jeddah, Saudi Arabia. Data on demographic characteristics, history of NSIs, nature of work, compliance with infection control protocols, and knowledge of infection control procedures and disease transmission were collected using a self-administered questionnaire. Logistic regression analysis was used to assess factors associated with NSIs; unadjusted and adjusted odds ratios (aORs) and their respective 95% confidence intervals (CIs) were computed.

**Results:**

About three in ten dental assistants experienced at least one NSI (29.8%, 95% CI 25.6–34.2%) in private dental clinics. Lack of adequate knowledge of infection control procedures and disease transmission, non-compliance with infection control protocol of vaccination against hepatitis B virus, and attending 12 or less number of patients daily were significantly associated with increased risk of NSIs (*p* ≤ 0.05); adjusted odds ratios (95% CI) were 1.87 (1.18–2.97), 1.89 (1.05–3.41), and 1.63 (1.03–2.56), respectively. In addition, dental assistants working in 45.8% of dental clinics that had no infection control unit were positively associated with higher NSI risk (aOR = 2.28, 95% CI 1.45–3.57).

**Conclusion:**

Our study reported the prevalent nature of NSIs among dental assistants in Saudi Arabia and identified key factors that could be targeted to mitigate this preventable condition. Dental assistants would benefit from proper training on infection control protocols and procedures.

## Background

Healthcare workers (HCWs) represent 12% of the global workforce [1, 2]. The risk of occupational exposure to infectious diseases is high among HCWs due to the nature of their work and proximity to infected patients rendering them susceptible to direct (e.g., through airborne transmission) or indirect (contamination of instruments or surfaces) transmission of infection.

Contaminated needlestick and sharp injuries (NSIs) are the most common sources of infection among HCWs [2]. Approximately 3 out of 35 million HCWs worldwide experience needlestick injuries (NSIs) annually, exposing them to blood-borne pathogens [2, 3]. Although as many as 20 different pathogens could be transmitted by NSIs [4], hepatitis B virus (HBV), hepatitis C virus (HCV), and human immunodeficiency virus (HIV) constitute the majority of blood-borne infections transmitted post-injury. Percutaneous occupational exposure accounts to approximately 37% of HBV, 39% of HCV, and 4.4% of HIV cases among HCWs [5]. As dental practice involves regular use of sharp instruments, and exposure to saliva, blood, and naturally occurring oral bacterial flora [6], dentists were more prone to hepatitis B infection than the general adult population [7]. In addition to the possibility of transmission of infection, consequences of NSI affect the daily life of HCWs causing stress and anxiety and in extreme cases disability and mortality [8–10].

Although previous studies reported some factors associated with increased risk of NSIs among HCWs, the reported factors varied by profession and geographic region. For example, experience of the practitioner, number of patients attended on a daily basis, recapping needles, knowledge of infectious diseases, and compliance with infection control protocols were among personal factors linked to NSIs among dentists in Taiwan [11]. However, in another study among Mangolian healthcare workers [12], longer work hours and number of injections administered in a day were associated with higher risk. Procedures such as needle recapping were predominant among healthcare workers experiencing NSI in Kabul [13]. Perceptions of medical students towards NSI risk influenced their risk of injury in Serbia [14]. The World Health Organization (WHO) emphasizes the implementation of universal infection prevention measures and the need for properly training HCWs in reducing the prevalence of NSIs in a healthcare setting [15].

The dearth of literature on NSI risk among dental assistants in Saudi Arabia and rest of the world, especially in often overlooked private sector, prompted us to address this important research gap. For the first time in Saudi Arabia, the present study determined the burden of NSIs among dental assistants and identified associated factors for NSIs from demographic characteristics, nature of work, compliance with various infection control protocols, and knowledge of infection control procedures and disease transmission.

## Methods

This cross-sectional study was conducted on a sample of dental assistants working in private dental clinics in Jeddah, Saudi Arabia, between June and September 2017 to determine the prevalence of NSIs and their associated factors. Dental assistants comprised of all qualified healthcare workers, such as nurses that assisted dentists with patient management in dental clinics.

The study participants were recruited from eight randomly selected private dental clinics from each of the five regions of Jeddah, including eastern, western, central, northern, and southern areas. All dental assistants who agreed to participate in the study were included without any exceptions. Informed consent was obtained from all the participants, and data confidentiality has been maintained.

### Study questionnaire

The study questionnaire was developed in English language based on previous literature [5, 10–12, 15, 16]. The face validity of the questionnaire and feasibility were established prior to data collection. We collected data on age, gender, years of experience (3 years or less/more than 3 years), nature of the clinic worked (e.g., endodontics, prosthodontics, and surgery), worked in more than one clinic (1/2/3 or more), duration of workday (8 h or less/more than 8 h), average number of patients attended per day (12 or less/more than 12), history of needlestick and sharp injuries (yes/no), frequency of past injuries, instrument causing the injury, procedure causing the injury, status of instrument contamination, injury reporting (yes/no), test for infection (yes/no), and receipt of post-exposure prophylaxis (yes/no).

Information on performing various clinical procedures (yes/no), such as needle recapping, needle exchange, transmitting instruments, picking up instruments, washing sharp instruments, sharp instrument disposal, local anesthesia administration, wound suturing, and scaling every working day, was also obtained. Dental assistants who answered yes to six or more questions were considered to be preforming high-risk procedures, and the rest of them were deemed performing low-risk procedures daily. We used this criterion to create a new reliable composite variable for high-risk procedures (high/low) in our analyses (Cronbach’s alpha = 0.79).

Compliance of dental assistants to various infection control protocols (yes/no), such as vaccination of dental assistants against HBV; use of personal protective equipment like gloves, face masks, and gowns; protective glasses and face shields; protective wrap; needle recapping using one-hand technique; using disposable burs; and proper waste disposal procedure, was also obtained.

Level of knowledge on infection control and transmission of infectious diseases among dental assistants was assessed based on responses to questions seeking information on whether the assistants knew the temperature and time required for complete sterilization; knowing the risk of transmission of HBV, HBC, and HIV; HBV viability on clinic surfaces; and whether they knew which infectious disease has the highest rate of transmission in saliva. A composite variable for knowledge was constructed based on the final score calculated by coding the correct answers with 1, and adding up all seven responses to obtain the final score. Participants who scored 3 or less were considered to have poor knowledge level, whereas participants who scored more than 3 were classified as having good level of knowledge. The composite variable for level of knowledge was reliable (Cronbach’s alpha = 0.65) in representing knowledge factors in our analysis. Participants also provided data on whether they had formal infection control training (yes/no) and whether the dental hospital they worked in had an infection control unit (yes/no).

### Statistical analysis

The data were analyzed using SAS software version 9.4 (SAS Institute Inc., Cary, NC, USA). Frequency counts and percentages for categorical variables, and mean and standard deviation (SD) for continuous variable were computed. The Clopper-Pearson exact tests were used to construct 95% confidence intervals for proportion. Pearson’s chi-squared test (or Fisher’s exact tests for smaller samples) and *p* values were used to assess the independence of various sample characteristics by NSI experience (yes/no).

To determine associated factors for experiencing NSIs, the unadjusted odd ratios (uORs), adjusted odds ratios (aORs), and their respective 95% confidence intervals (95% CIs) were computed using logistic regression analysis. The study subjects missing data on characteristics considered in our models were excluded in the analyses. The calibration of multivariate model was assessed using the Hosmer and Lemeshow goodness-of-fit test, and multicollinearity was assessed based on collinearity indices, Eigen values, and variable decomposition proportions. The statistical significance was based on a *p* value of 0.05 or less.

## Results

Our analysis was based on a sample of 450 dental assistants who participated in our survey (among 500 eligible subjects invited to participate). The participants were predominantly female (96%) with an average age of 31.1 years (SD ± 6.9). A total of 134 participants experienced needlestick and sharp injuries (29.8%, 95% CI 25.6–34.2%). A significant number of these injuries were caused by needles (53%), mainly during recapping (23%). Approximately 63% of the NSIs were not reported, 35% underwent testing post-injury, and 19% of the dental assistants received post-exposure prophylaxis for their injury.

The descriptive statistics for various characteristics of study population were reported in Table 1 as frequencies and percentages. About 50% of study subjects were working in three or more clinical sub-specialties in a dental clinic. Results also revealed that the majority of subjects worked 8 h or less in a day (76%), attended 12 patients or less (57.1%), had less than 3 years of work experience (55.1%), and had poor knowledge of infection control and disease transmission process (58%). Experiencing NSIs in a dental clinic was dependent on workers receiving anti-HBV vaccination and the presence of infection control unit in a dental clinic (*p* < 0.05).

**Table 1:** Descriptive data of study sample by history of needlestick and sharp injury (NSI) experience

Characteristic	Category	Number of subjects (%)			*p* value*
		All (*n* = 450)	History of NSI		
			Yes (*n* = 134)	No (*n* = 316)	
Number of clinics worked	One	165 (36.7)	46 (34.3)	119 (37.7)	0.3359
	Two	58 (12.9)	22 (16.4)	36 (11.4)	
	3 or more	227 (50.4)	66 (49.3)	161 (50.9)	
Daily working hours	> 8 h	108 (24.0)	25 (18.7)	83 (26.3)	0.0839
	8 h or less	342 (76.0)	109 (81.3)	233 (73.7)	
Number of patients attended per day	> 12	193 (42.9)	48 (35.8)	145 (45.9)	*0.0485*
	12 or less	257 (57.1)	86 (64.2)	171 (54.1)	
Years of experience	3 years or less	248 (55.1)	66 (49.3)	182 (57.6)	0.1038
	> 3 years	202 (44.9)	68 (50.7)	134 (42.4)	
Performing procedures carrying high risk	No	168 (37.3)	46 (34.3)	122 (38.6)	0.3908
	Yes	282 (62.7)	88 (65.7)	194 (61.4)	
Anti-hepatitis B vaccination	No	64 (14.2)	30 (22.4)	34 (10.8)	*0.0012*
	Yes	386 (85.8)	104 (77.6)	282 (89.2)	
Wore gowns, mask, and gloves	No	74 (16.4)	29 (21.6)	45 (14.2)	0.0528
	Yes	376 (83.6)	105 (78.4)	271 (85.8)	
Used eye and facial protection	No	83 (18.4)	32 (23.9)	51 (16.1)	0.0528
	Yes	367 (81.6)	102 (76.1)	265 (83.9)	
Used protective films	No	65 (14.4)	22 (16.4)	43 (13.6)	0.4381
	Yes	385 (85.6)	112 (83.6)	273 (86.4)	
Used disposable burs	No	197 (43.8)	67 (50.0)	130 (41.1)	0.0832
	Yes	253 (56.2)	67 (50.0)	186 (58.9)	
Used high-volume suction	No	41 (9.1)	16 (11.9)	25 (7.9)	0.1744
	Yes	409 (90.9)	118 (88.1)	291 (92.1)	
Recapped needles by one-hand technique	No	32 (7.1)	14 (10.4)	18 (5.7)	0.0729
	Yes	418 (92.9)	120 (89.6)	298 (94.3)	
Knowledge of infection control procedures and disease transmission	Good	189 (42.0)	41 (30.6)	148 (46.8)	*0.0014*
	Poor	261 (58.0)	93 (69.4)	168 (53.2)	
Formal infection control training	No	119 (26.4)	36 (26.9)	83 (26.3)	0.8950
	Yes	331 (73.6)	98 (73.1)	233 (73.7)	
Infection control unit in the clinic	No	206 (45.8)	82 (61.2)	124 (39.2)	*< 0.0001*
	Yes	234 (52.0)	52 (38.8)	182 (57.6)	
	Missing	10 (2.2)	0 (0.0)	10 (3.2)	

*p value was based on Pearson’s chi-squared test to evaluate the independence of sample characteristic and NSI experience

Table 2 shows the unadjusted (uOR) and adjusted odds ratios (aOR) and their 95% confidence intervals (CIs) for the association between various population characteristics and NSI experience. Dental assistants with poor knowledge of infection control and disease transmission process experienced 1.9-fold higher risk of NSIs than those with good knowledge (aOR = 1.87, 95% CI 1.18–2.97). Lack of infection control unit in the dental clinic was significantly associated with NSI experience in our sample (aOR = 2.28, 95% CI 1.45–3.57). Subjects that were not vaccinated for HBV and attending 12 patients or less in a day were significantly associated with higher NSI experience; adjusted odds ratios (95% CI) were 1.89 (1.05–3.41) and 1.63 (1.03–2.56), respectively. The final model was well calibrated (*p* = 0.5451, Hosmer and Lemeshow goodness-of-fit test), and multicollinearity was not an issue.

**Table 2:** Unadjusted (uOR) and adjusted odds ratios (aOR) and their respective 95% confidence intervals (95% CIs) for the relationship between characteristics of study population and needlestick and sharp injury (NSI) experience

Characteristic	Category	Unadjusted odds ratios			Adjusted odds ratios		
		uOR	95% CI	*p* value	aOR	95% CI	*p* value
Number of clinics worked	One^†^	1.00			1.00		
	Two	1.47	0.78–2.77	0.2283	1.26	0.64–2.49	0.5009
	3 or more	1.00	0.64–1.57	0.9943	1.00	0.61–1.62	0.9877
Daily working hours	> 8 h^†^	1.00			1.00		
	8 h or less	1.60	0.97–2.64	0.0685	1.46	0.82–2.58	0.1949
Number of patients attended per day	> 12^†^	1.00			1.00		
	12 or less	1.55	1.02–2.36	0.0398*	1.63	1.03–2.56	0.0358*
Years of experience	> 3 years	1.39	0.93–2.10	0.1095	1.48	0.95–2.31	0.0853
	3 years or less^†^	1.00			1.00		
Performing procedures carrying high risk	No^†^	1.00			1.00		
	Yes	1.14	0.74–1.74	0.5571	1.29	0.82–2.04	0.2646
Anti-hepatitis B vaccination	No	2.31	1.34–3.96	0.0024*	1.89	1.05–3.41	0.0333*
	Yes^†^	1.00			1.00		
Wore gowns, mask, and gloves	No^†^	1.60	0.95–2.69	0.0750	1.37	0.67–2.79	0.3886
	Yes^†^	1.00			1.00		
Used eye and facial protection	No	1.61	0.97–2.65	0.0629	1.14	0.60–2.18	0.6834
	Yes^†^	1.00			1.00		
Used protective films	No	1.20	0.69–2.10	0.5202	0.83	0.43–1.58	0.5642
	Yes^†^	1.00			1.00		
Used disposable burs	No	1.37	0.91–2.06	0.1282	1.20	0.77–1.88	0.4239
	Yes^†^	1.00			1.00		
Used high-volume suction	No	1.59	0.82–3.11	0.1717	1.14	0.54–2.42	0.7288
	Yes^†^	1.00			1.00		
Recapped needles by one-hand technique	No	1.87	0.90–3.87	0.0938	1.59	0.69–3.62	0.2732
	Yes^†^	1.00			1.00		
Knowledge of infection control procedures and disease transmission	Good^†^	1.00			1.00		
	Poor	2.10	1.36–3.23	0.0007*	1.87	1.18–2.97	0.0082*
Formal infection control training	No	1.15	0.72–1.83)	0.5500	0.86	0.52–1.45	0.5778
	Yes^†^	1.00			1.00		
Infection control unit in the clinic	No	2.31	1.53–3.51	< 0.0001*	2.28	1.45–3.57	0.0003*
	Yes^†^	1.00			1.00		

*p values reported were significant based on significance level of 0.05

^†^Reference category for the variable

## Discussion

The present study determined that approximately 30% of dental assistants working in private dental clinics in Saudi Arabia experienced at least one NSI during their working life. We identified several key factors associated with NSI experience among dental assistants, including vaccination against HBV infection, attending12 patients or less in a day, poor knowledge of infection control and disease transmission process, and lack of infection control unit in dental clinic.

The prevalence of NSI experience among dental assistants in Saudi Arabia was similar to those in Iran (31%) [17], but considerably lower than the prevalence (75%) reported in Germany [18]. Our results were consistent with prior studies that reported needles as the main source of NSI [13, 18–22]. In a previous study conducted among nurses working in a regular university hospital in Saudi Arabia [23], 45% of nurses experienced an NSI indicating the prevalent nature of this preventable condition in Saudi Arabia [23].

Forty-two percent of dental assistants in the current study reported good level of knowledge by answering many questions in the survey correctly. This percentage was somewhat similar to that reported among dental assistants in Iran, where dentists’ knowledge score was 4.88 out of 10 [17]. In the current study, increased level of knowledge of infectious disease transmission was found to be significantly associated with lower risks of NSIs. These results were similar to those reported in a study of Taiwan participants [11] which showed that those who lacked knowledge about oral signs of HIV were at a 60% increased risk of suffering a NSI. The present study evaluated knowledge of a number of variables such as knowledge of temperature and time required for complete sterilization, and knowledge of the risk of transmission of HBV, HVC, and HIV. These reflect increased awareness of knowledgeable dental assistants regarding factors influencing infection at dental clinics, which might have contributed to their decreased risk of NSI.

Treating a lower number of patients per day was found to be positively associated with higher NSIs. This can be attributed to the fact that those treating more patients may have accumulated more experience in handling devices and efficiently performing the different tasks without enduring more risk of NSI. Our findings are in contrast to the findings of the study in Taiwan, in which dentists were 3.57 times more likely to suffer an NSI when treating more than 30 patients [11]. In addition, Ebrahimi et al. reported that there was no relationship between treating the number of patents and the risk of NSI [17]. However, in agreement with our current study, another study conducted in Germany reported that treating more patients was associated with lower risk [18]. Although the collective evidence has been inconsistent, the varying risk observed among different professions and geographic regions warrants further investigation of the role of patient load on NSI risk.

The risk of NSIs was consistently higher among dental workers, in several countries, who exhibited poor compliance to infection control protocols [11, 12, 24–26]. Universal infection control protocols [15] were developed based on evidence of effectiveness to prevent incidents of infectious disease transmission and to protect the patient and healthcare workers, so it is naturally presumable that a negative relationship exists between compliance and injuries. Assistants adhering to protocols also indicate that they are more cautious; thus, lower numbers of injuries will affect them. It is noteworthy that vaccination against HBV was the predominant infection control protocol influencing lower risk of NSI experience among dental assistants in our study. In a study of the global risk of hepatitis B among healthcare workers, the dental community was found to have the highest infection risk of all healthcare personnel [27]. Given this finding, and significant burden of HBV infection in the Far East, Middle East, Africa, and parts of South America (HBV surface antigen rates ranging between 8 and 15%), HBV vaccination among HCWs in general and dental staff in particular is an important preventive measure [28]. Therefore, anti-HBV vaccine should be made mandatory for all healthcare workers in both public and private care settings in Saudi Arabia.

It is interesting to note that lack of infection control unit within the dental clinic was associated with increased risk of NSI. This can be also explained within the scope of compliance to infection control protocols. Not having an infection control unit may be associated with overall lack of training or low levels of awareness of the dental staff to the issues of infection control and occupational safety or both. This is in contrast to other clinics which may have such a unit and thereby have better awareness of occupational safety.

The present study addressed the risk of NSIs among dental assistants for the first time in Saudi Arabia. Unlike previous studies that mainly focused on NSI risk among HCWs in public sector [29–31], our study contributed to much needed background data in private care settings. Furthermore, we assessed NSI risk for wide-ranging associated factors to inform public health efforts directed at mitigating NSIs in HCWs. The current study did not explore the prevalence and factors leading to NSI among dentists. This occupational group can be at high risk due to the nature of their job including frequent handling of needles and other sharp objects. A recent study conducted in three types of dental clinics in Riyadh, Saudi Arabia [32], found the prevalence NSI among dentists of about 21%. This was slightly lower than the prevalence in dental assistants of 29% reported in the current study. It is expected that risk factors such as education, work load, training, and other factors differ between dentists and dental assistants. Indeed, results from the study [32] suggested that more professional experience and greater compliance with infection control procedures were associated with lower risk of NSI.

This study was subjected to some limitations. Foremost, the readers should refrain from drawing causal inferences due to the cross-sectional study design. However our findings contributed to the knowledge of NSIs in dental assistants and have the potential to inform future studies in this vulnerable workforce. Although one should be cautious about generalizing findings from one city to the entire country, we expect our results to be representative of private dental sector in the country owing to the diversity of clinic types included, governance structure, and cultural homogeneity in Saudi population. Self-reported data was subject to recall bias, but we would expect minimal effect on our results owing to routine and current practices tested in the questionnaire.

Another limitation that this study did not explore was the prevalence and associated factors of NSI among dentists due to limited resources. Such information would be valuable in comparing the risk between the two groups in the dental occupation and may be explored in future studies.

## Conclusions

About three in ten dental assistants working in private dental clinics experienced at least one NSI during their lifetime, indicating the prevalent nature of this preventable condition in Saudi Arabia. Our study highlighted NSI risk among participants lacking proper knowledge on infection control and disease transmission in dental clinic setting, and non-compliance to hepatitis B vaccination. This warrants proper training of dental personnel in infection control protocols at the workplace [33, 34]. Patient load as a factor influencing NSI should be further explored in future research owing to inconsistent evidence worldwide. The positive effect of infection control unit in reducing the experience of NSIs should encourage dental clinics towards the establishment of independent infection control units in their facility. Overall, our study contributed to the knowledge of NSIs among dental assistants in often overlooked private care facilities.

## Data Availability

The datasets used and/or analyzed during the current study are available from the corresponding author on reasonable request.

## Abbreviations

aOR: Adjusted odds ratio
CI: Confidence interval
HBV: Hepatitis B virus
HCV: Hepatitis C virus
HCWs: Healthcare workers
HIV: Human immunodeficiency virus
NSI: Needlestick and sharp injury
SD: Standard deviation
uOR: Unadjusted odds ratio
WHO: World Health Organization
